# Supplementary material for: Identification of photoperiod-induced specific miRNAs in the adrenal glands of Sunite sheep (Ovis aries)
Source: Front Vet Sci. 2022 Jul 22;9:888207. doi: 10.3389/fvets.2022.888207 (PMC9354845; doi:10.3389/fvets.2022.888207)
Supplement: Supplementary file 1 [file Data_Sheet_1.ZIP › Supplementary file/miRDeep2 file.zip!/miRDeep2 file/Know_Structure/SP42A2/oar-let-7i.pdf]

miRBase precursor : oar-let-7i
Total read count : 432675
oar-let-7i read count : 432420
remaining reads : 255

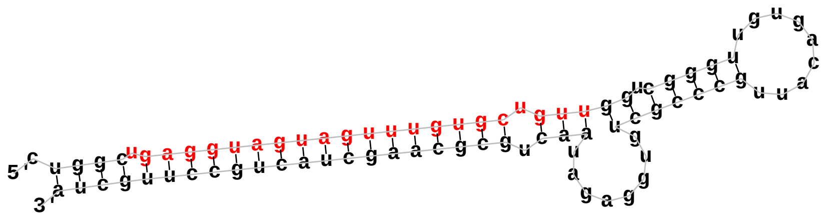

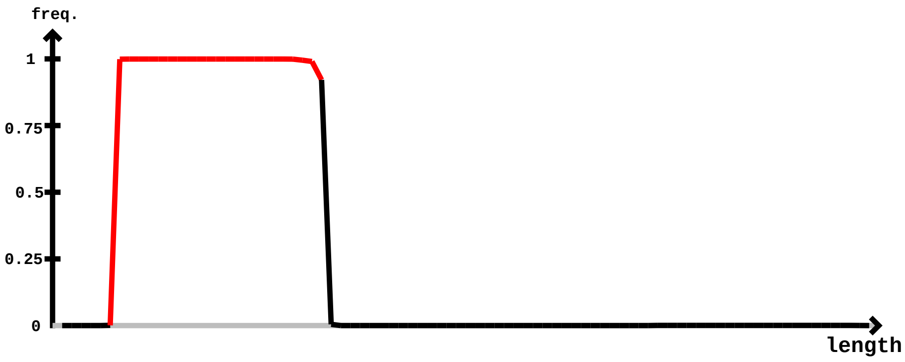

oar-let-7i

5'- cuggcugagguaguaguuugugcuguuggucggguugugacauugcccgcuguggagauaacugcgcaagcuacugccuugcua -3'	exp		
.((((.(((((((((((((((((.(((((.(((((.........)))))))........))).)))))))))))))))))))))	reads	mm	sample
....cugagguaguaguuugu...	2	0	seq
....cugagguaguagAuugu...	2	1	seq
....cugagguaguaguuugugcu..	2	0	seq
....cugagguaguaguuugugcug...	3	0	seq
....cugagguaguaguuugugcugu..	40	0	seq
....cugagguaguaguuugugcuguu...	346	0	seq
....cugagguaguaguuugugcuguug..	1	0	seq
.....ugagguaguaguuugug..	20	0	seq
.....ugagguaguaguuugugA...	17	1	seq
.....ugUgguaguaguuugugc...	1	1	seq
.....ugagguaguaguuugugG...	1	1	seq
.....ugagguaguaguuugugc...	111	0	seq
.....ugagguaguaguuugugcu..	1705	0	seq
.....ugagguaguaguuugugcug...	2119	0	seq
.....ugagguaguaguuugugcugu..	29778	0	seq
.....ugagguaguaguuugugcuguu...	396021	0	seq
.....ugagguaguaguuugugcuguug..	2012	0	seq
.....ugagguaguaguuugugcuguugg...	8	0	seq
.....ugagguaguaguuugugcuguuggu..	1	0	seq
.....ugagguaguaguuugugcuguugguc...	1	0	seq
......gagguaguaguuugugcugu..	6	0	seq
......gagguaguaguuugugcuguu...	217	0	seq
......gagguaguaguuugugcuguug..	2	0	seq
.......agguaguaguuugugcuguu...	1	0	seq
........Uguaguaguuugugcuguu...	1	1	seq
........gguaguaguuugugcuguu...	1	0	seq
..........uaguaguuugugcuguu...	1	0	seq
....cugcgcaagcuacugccuu....	1	0	seq
....cugcgcaagcuacugccuug...	13	0	seq
....cugcgcaagcuacugccuugc..	94	0	seq
....cugcgcaagcuacugccuugcu.	144	0	seq
....cugcgcaagcuacugccuugcua	1	0	seq
....ugcgcaagcuacugccuugc..	2	0	seq
